# Short (6 mm) and Regular Dental Implants in the Posterior Maxilla–7-Years Follow-up Study

**DOI:** 10.3390/jcm10050940

**Published:** 2021-03-01

**Authors:** Jakub Hadzik, Paweł Kubasiewicz-Ross, Izabela Nawrot-Hadzik, Tomasz Gedrange, Artur Pitułaj, Marzena Dominiak

**Affiliations:** 1 Department of Dental Surgery, Wrocław Medical University, 50-425 Wrocław, Poland; pawel.kubasiewicz-ross@umed.wroc.pl (P.K.-R.); artur.pitulaj@umed.wroc.pl (A.P.); marzena.dominiak@umed.wroc.pl (M.D.); 2 Department of Pharmaceutical Biology and Botany, Wrocław Medical University, 50-367 Wrocław, Poland; izabela.nawrot-hadzik@umed.wroc.pl; 3 Department of Orthodontics, Dresden University of Technology, 01069 Dresden, Germany; tomasz.gedrange1@tu-dresden.de

**Keywords:** dental implants, short implants, sinus lift, regular implants

## Abstract

Short 6 mm dental implants are considered as an alternative to the maxillary sinus elevation and bone augmentation procedure where there is a reduced alveolar ridge height. The aim of this study was to compare the implant survival rate between short dental implants (6 mm) and regular length implants (11–13 mm) when placed in combination with bone grafting and loaded with a single non splinted crown, seven years after placing the implant. It was conducted as a controlled clinical study of 30 patients with partial edentulism in the posterior maxilla. The protocol included radiological and clinical evaluation of the C/I ratio (length of the superstructure divided by the length of the implant crestal part), marginal bone level (MBL), ultrasonography measurement of soft tissue surrounding implant (STT), patient-reported outcomes, and biological and technical complications. A total number of 28 implants (93%) remained integrated during follow-up period. MBL of 0.50 and 0.52 mm was observed for short implants and regular implants, respectively. MBL was checked for correlation with STT, and a negative correlation was found between MBL: STT. Our study has demonstrated a significantly lower implant survival rate for short implants compared to regular implants (87% compared to 100%). Despite the loss of several implants, good clinical results were achieved in the remaining implants in both groups. It is, therefore, worth considering short implants as an alternative to regular implants with a sinus lift surgery.

## 1. Introduction

Implant treatment can be performed in the edentulous maxilla only when there is an adequate amount and good quality of bone tissue. After the tooth extraction, the bony socket undergoes a series of adaptive changes, both vertical and horizontal, to reduce bone height [1,2]. When there is advanced bone loss, and the extensive maxillary sinus is present, it is impossible to place the implant without bone augmentation in the maxillary sinus. Maxillary sinus floor augmentation surgery (MSFA) for regular length implant placement is a well-recognized and well-documented medical procedure where the survival rate is typically much greater than 90% in long-term evaluations [3]. Sinus lift procedure, first described by Boyne and James, has been redesigned by Tatum [4,5]. This technique creates additional space between the maxillary alveolar process and the elevated Schneiderian membrane which is filled with various graft materials to maintain adequate space for new bone formation [6]. Up to the present day, many modifications using different materials and techniques have been described, however, high skills of the operator are still required and a number of complications may still occur, including a higher risk of surgical site infections, graft failure, and post-operative sinusitis [7,8,9,10].

The indisputable progress and improvement of the implant surface enabled the reduction of the length of the implant, while maintaining proper stability and functionality. Implants with reduced length (short implants) have been introduced to fit horizontally reduced alveolar bone and to avoid the need for a sinus augmentation procedure. A clear definition of short dental implants has not emerged in the literature and there is still some controversy over their definition. For the purpose of this study, implants with a designed intrabony length of 6 mm were considered short.

Iezzi et al., in a recent systematic review and meta-analysis of short implants in the posterior region, have found that short implants (<7, <5 mm) show well-documented clinical performance and effective survival rate [11]. Similarly, Torres-Alemany et al., in a recent systematic review, focused on short implant crown-to-implant ratio and its impact on implant survival. These authors have found that short implants do not seem to have a significant influence on marginal bone loss or the survival rate of implants [12].

The primary aim of the presented study was to compare the implant survival rate between short dental implants (6 mm) and regular length implants (11–13 mm) placed in combination with bone grafting loaded with a single non splinted crown seven years after implant placement. The secondary aim of the study included radiological and clinical evaluation of the marginal bone level (MBL), patient-reported outcomes, and biological and technical complications. 

## 2. Material and Methods

### 2.1. Study Design

The presented study was designed as a prospective randomized controlled study (RCT). The study was performed in Wroclaw Medical University Dental Clinical and Teaching facility. The study protocol of the original RCT was approved by a local ethical committee (registration number 587/2012) and conducted in 2012–2013 the RCT as a clinical trial was registered under the clinical trial registration number NCT03471000 (ClinicalTrials.gov). All patients gave two written consents: The first was general consent to have dental implants placed, and the other consent involved participation in the study. The study has been conducted in full compliance with the Declaration of Helsinki. A call for seven years follow-up during this prospective clinical trial with an updated and modernized protocol required a new bioethics committee approval that was granted (registration number 572/2018).

Details on the specific clinical procedures as well as the three-year marginal bone loss (MBL) data were reported in earlier studies by Hadzik et al. [13,14]. In brief, 30 patients (20 females, 10 males, average age at the time of surgery 45.5 years) with partial edentulism in the posterior maxilla (ridge height between 6 and 7 mm and ridge width ≥6 mm) were recruited according to a randomization protocol and treated with either short implants (group short: G1; 6 mm) or regular implants in combination with a lateral window sinus floor elevation procedure (group graft: G2; 11–13 mm). In the previous study, the three years MBL was reported 0.22 ± 0.46 mm 0.34 ± 0.24 mm for regular and short implants, respectively. Implant survival rate was 100% for both groups. One implant was placed in each patient and each implant was loaded with a single non splinted crown. All implants were placed in posterior maxilla. 

Inclusion criteria were:

- Adult patients (>18 years old)
- Single missing tooth (first or second molar) in the lateral aspect of the maxilla to be replaced with a single implant-supported crown at the beginning of the study.
- Tooth loss due to caries, root canal treatment failure, no history of periodontal disease
- Minimal height of the alveolar ridge of 6 mm in the region of the implant insertion in the pre-surgical qualification
- Minimal width of the alveolar ridge of 6–7 mm in the region of interest
- API £35 (approximal plaque index)
- PI £25 (plaque index)

Exclusion Criteria were:

- Previous graft procedures in the area of interest
- Systemic or local diseases that could compromise healing or osteointegration
- Heavy smokers
- Patients with bruxism

**Figure 1:**
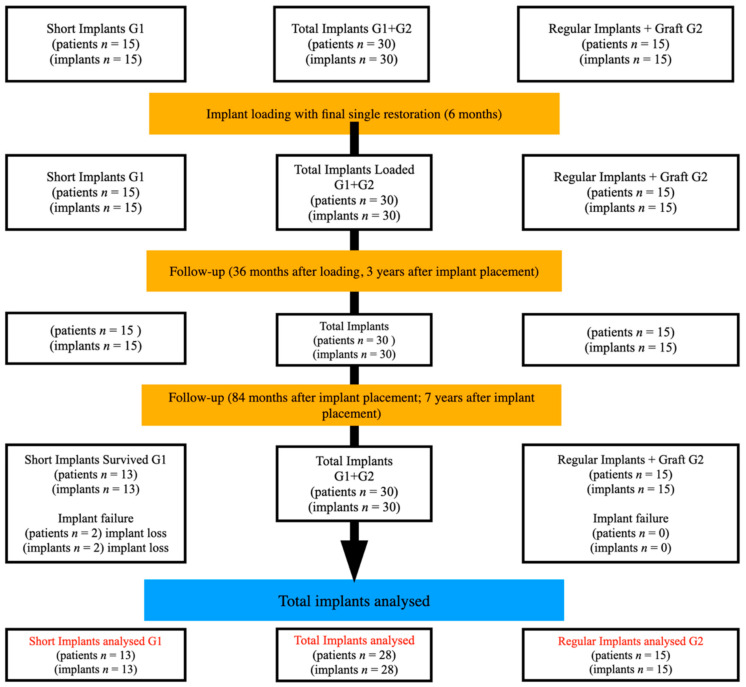
Flow chart of study schedule and timeline.

A study schedule and timeline are presented in Figure 1. 

### 2.2. Clinical Procedures: Randomization, Surgery, Implant Loading

**Figure 2:**
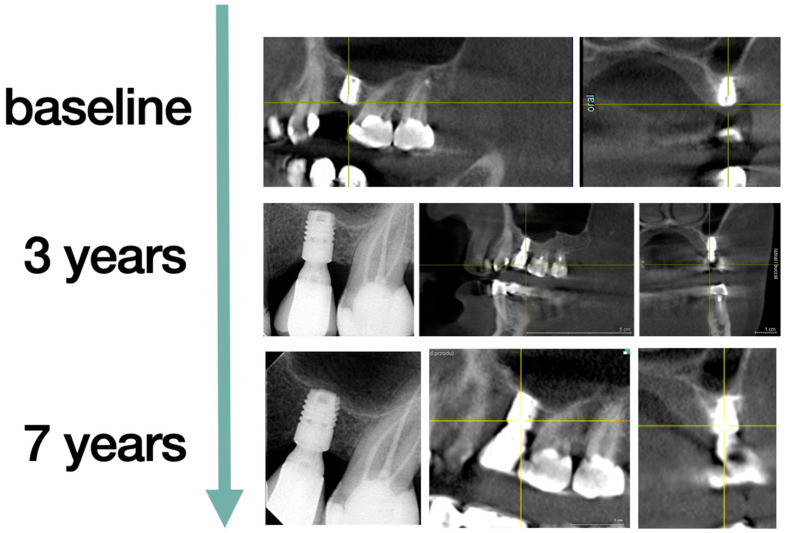
Short implants. The same patient from baseline to seven years, implant tooth 26, follow-up. Periapical X-ray and Cone Beam Computed Tomography (CBCT) are presented.

**Figure 3:**
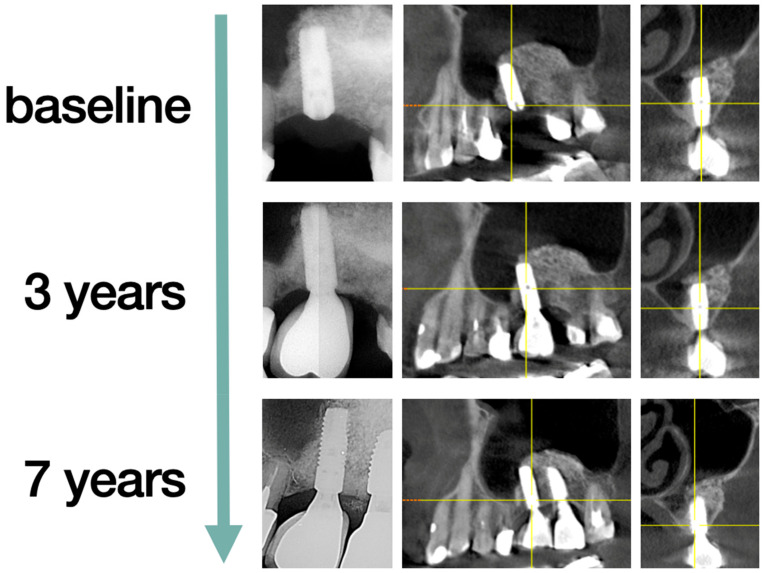
Regular implants. The same patient from baseline to seven years, implant tooth 26, follow-up. The second implant was placed after tooth 27 was lost and restored with a second single implant crown and is not included in the measurements. Periapical X-ray and CBCT are presented.

The randomization was performed on the day of surgery by drawing the ticket out of the envelope. The patients were then divided into two groups according to the method of treatment provided. The first group (G1) of n = 15 patients had two piece short-length implants (OsseoSpeed^TM^ L6mm Ø4mm, Dentsply Sirona Implants, Mölndal, Sweden) placed without the sinus lift and augmentation procedure (Figure 2). The second group (G2) of n = 15 patients had regular two piece dental implants (OsseoSpeed^TM^ L11 Ø4 mm and L13 Ø4 mm, Dentsply Sirona Implants, Mölndal, Sweden) placed, preceded by the sinus lift procedure from a lateral window approach with the application of the Xenogeneic bone graft Geistlich Bio-Oss^®^ (Geistlich AG, Wolhusen, Switzerland) (Figure 3). 

Six months after the implant placement, an impression of the implant was made following final restoration. All implants were loaded with single porcelain fused to metal restoration (PFM) non-splinted prosthetic crown, which was cemented with a semipermanent implantlink^®^ semi cement (Detax, Ettlingen, Germany) on an implant round titanium abutments for cement-retained restorations (Dentsply Sirona Implants, Mölndal, Sweden). The occluding relations were controlled using articulating paper (Bausch^®^, Cologne, Germany) with a thickness of 200, 80, and 8 μm.

### 2.3. Follow-up

Having finished the period of a 36-month observation, the patients were scheduled for regular (once a year) follow-up visits in their place of residence for their convenience. Two of the patients totally neglected the control visits and maintenance. All patients were called for a follow-up visit after seven years of implant placement. A clinical trial surgery and a three-year and seven years follow-up clinical appointment were conducted by the same principal investigator (JH). All patients who participated in the original study and had implants placed were included in the follow-up. 

### 2.4. Clinical Outcome

Clinical evaluation included bleeding on probing (BOP) and probing depths (PD) at four sites (mesial, distal, buccal, lingual) and technical or biological complications, such as periimplantitis. Peri-implantitis, according to Derks et al., was defined as implants demonstrating bleeding on probing/suppuration and bone loss >2 mm [15]. The height of the keratinized tissue (HKT) was clinically measured with a dental periodontal probe with a millimeter scale. Additionally, during the last clinical examination, the soft tissue thickness (STT) in the implant surrounding site was examined using the Pirop^®^ (Echoson, Puławy, Poland) ultrasound device.

### 2.5. Marginal Bone Level

**Figure 4:**
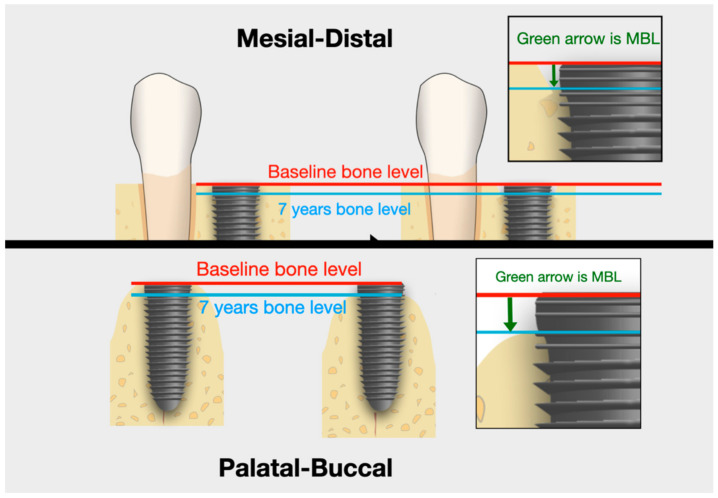
Marginal bone level (MBL) measuring points on CBCT were located around the implant platform (four points around each mesial, distal, buccal, and palatal).

Before the surgery, after 36 months and after seven years the CBCT (Cone Beam Computed Tomography) (Galileos D3437, Sirona Dental Systems GmbH, Bensheim, Germany), and dental X-ray (Visualix eHD, Gendex Dental Systems, Des Plaines, IL, USA) examinations were performed to assess the marginal bone loss (MBL). The MBL measurement was based on the CBCT image and with the usage of a standard dental periapical x-ray done with straight angle technique with a standard (non-individualized) positioner. The CBCT image offers transrectal views so the measurement can be made around the implant. The authors have chosen the CBCT evaluation since it can show the bone loss in the buccal/palatal aspect that might not be visible on periapical 2D x-ray. Since the implants were inserted at a bone level, the position of the implant neck above the alveolar crest level was taken as a reference point for all of the MBL measurements. The measuring points on CBCT were located around the implant (four points around each mesial, distal, buccal, and palatal) and the mean values were calculated (Figure 4). To indicate the value in millimeters, in each case, the radiological measurement was calibrated with the previously known length of the implant.

### 2.6. Crown-to-Implant Ratio (C/I Ratio)

**Figure 5:**
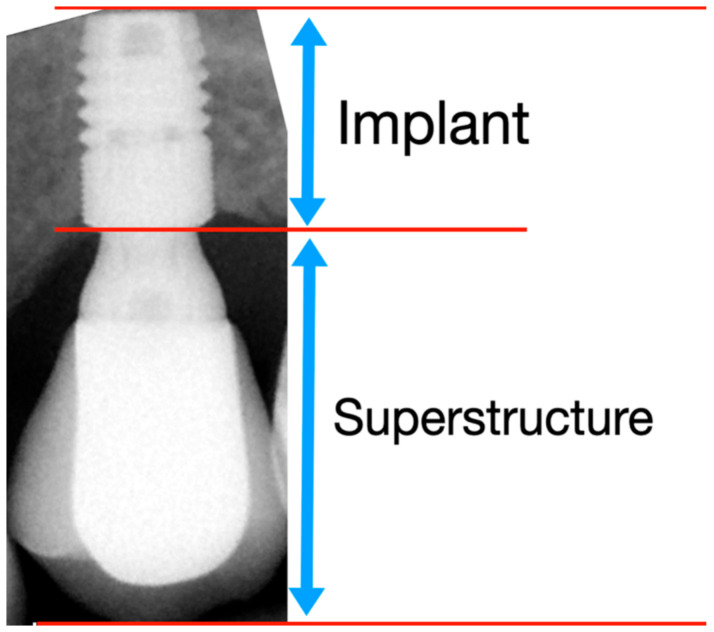
Crown-to-implant ratio was determined by dividing the length of the superstructure (crown and the abutment) by the length of the implant.

The crown-to-implant ratio was calculated based on a RTG and determined by dividing the length of the superstructure (ceramic crown and the abutment) by the length of the implant that was placed crestally (Figure 5). 

### 2.7. Soft Tissue Measurement

In both groups of patients, the thickness of keratinized tissue and gingiva were measured. In the follow-up clinical protocol, compared to the previous one, authors have supplemented a STT examination using an ultrasound device. The data obtained in this way were then checked for correlation with HKT and MBL, defined as marginal bone loss around the implant. HKT was measured with a millimeter periodontal probe. STT was measured by using ultrasonography (USG) with a Pirop^®^ dental, ultrasound device according to a protocol developed by our research team and described previously in Puzio et al. [16].

### 2.8. Primary and Secondary Outcome Variables

The implant survival rate was defined as the primary outcome variable. Implant survival rate was calculated as the number of implants that remained integrated during the seven years follow-up period. In the case of implant loss, the patient’s data were excluded from the clinical and radiological data. Clinical and radiological results apply only to patients with an integrated implant during the observation period. Secondary outcome variables included PD at the implant site, BOP at the implant site, MBL, C/I ratio, HKT, STT, and biological and technical complications. 

### 2.9. Statistical Analysis

The statistical analysis was performed using GraphPad Prism 9 software [GraphPad Software, San Diego, CA, USA]. The data were tested to check the normal distribution by the Shapiro–Wilk and D’Agostino–Pearson omnibus tests. Depending on the criteria met, an appropriate test was selected for further analysis. A parametric and nonparametric statistical approach was applied depending on the nature of the data. Unpaired t-test for parametric, and a Mann–Whitney U Test for non-parametric data. Spearman’s rho test and Pearson correlation coefficients, depending on normal distribution, were used to measure correlation. All data were given as means ± standard deviation (SD). *p* < 0.05 was considered statistically significant. 

## 3. Results

Out of 30 implants placed in 30 patients who entered the study, 30 patients were recalled and re-examined with a modernized protocol. A total number of 28 implants (G1 n = 13, G2 n = 15) in 28 patients remained integrated after seven years follow-up. 

### 3.1. Implant Survival Rate

Two short implants were lost in the short implant group between year 5 and 7, contrary to all regular implants that remained in place. This resulted in 87% of implant survival rate in the short implant G1 group, and 100% implant survival rate in the regular implant G2 group. The case study and available data of two of the lost short implants will be presented in the discussion. Patient demographics and implant details are presented in Table 1.

### 3.2. Clinical Outcome Measures

**Figure 6:**
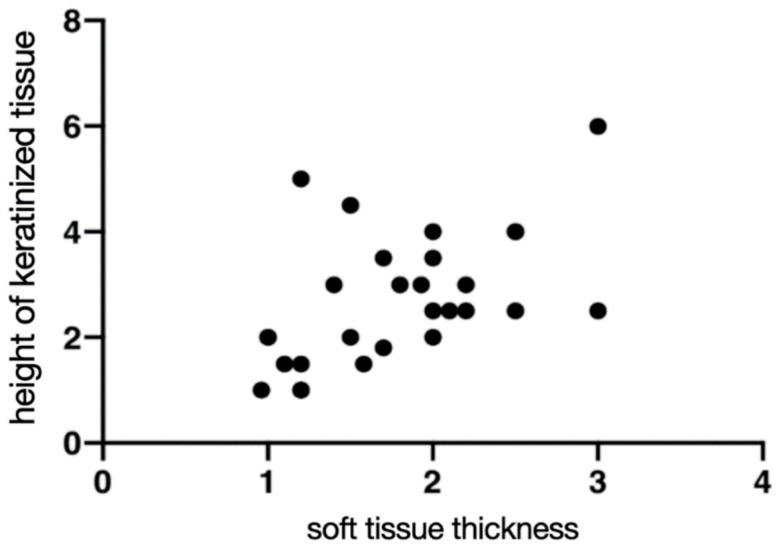
Correlation between the height of keratinized tissue (HKT) (seven years) and soft tissue thickness (STT) measured with Pirop^®^ ultrasonography device, presented value in (mm). Each dot represents an individual patient

PD, BOP, HKT, and STT are presented in Table 2. At seven years follow-up, there was no statistically significant difference in PD and BOP (*p* ≤ 00.5) between the groups. The biological complications, including peri-implant mucositis (BOP+ at implant site), were found in the short implant G1 group in 46.15% of patients and the regular implant G2 group in 60.00% of patients. The periimplantitis was reported when BOP+ and MBL > 2 mm were observed at the implant site at 0% in the short implant G1 and 13% in the regular G2. A positive correlation was found between HKT:STT (Table 2, Figure 6).

### 3.3. Crown to Implant Ratio (C/I Ratio)

The mean C/I ratio for short implants was 1.64 (ranging from 1.36 to 1.97) and for regular implants was 1.06 (ranging from 0.68 to 1.65). The differences between G1 and G2 C/I ratio were statistically significant. The C/I ratio was checked for correlation with the MBL data, no significant difference was found. Table 3 presents the values of C/I ratio for G1 and G2. 

### 3.4. Marginal Bone Loss

**Figure 7:**
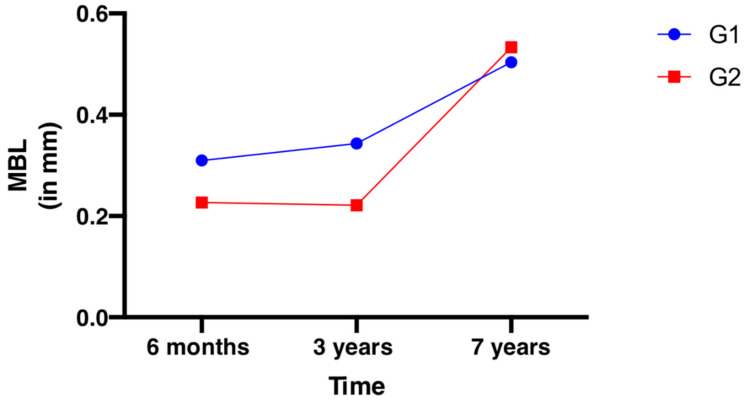
Change of marginal bone loss (MBL) over time. Six months, 36 months, seven years. For G1 and G2.

Since all of the implants were placed at the bone level, the level of 0 was taken as the initial value. The MBL reported in mm refers to a millimeter of bone loss compared to the initial value. At seven years, mean MBL of 0.50 and 0.52 mm was observed for G1 and G2, respectively, the difference was not statistically important. Data presented in Table 4 and Figure 7 present the MBL change over time for G1 and G2. The seven years data of MBL were checked for correlation with an HKT and STT. No correlation was found between MBL: HKT, and a negative correlation was observed between MBL:STT (Table 5 and Figure 7).

### 3.5. Technical Complications

During the study, a total number of five implant-level technical complications occurred (G1: 2; G2: 3). Chipping of the ceramic, de-cementation of the crown, and abutment screw loosening without the de-cementation of the crown were reported. The distribution of the technical complications among groups was 40% to 60% for short and regular implants, respectively. 

## 4. Discussion

The most relevant aspect of the study was the long-term evaluation of the effectiveness of treatment with short and long implants in the posterior maxilla. The parameters, such as implant survival rate, were evaluated, but also the overall cost/benefit of the described solutions was discussed. Attention was paid to the soft tissue parameters that, according to the literature, have an impact on the broadly understood success in implantological treatment. 

There are still some controversies over the definition of a short dental implant. According to Tawil and Younan [17], a dental intraosseous implant with a 10 mm intrabony part is considered short, whereas Nisand and Renouard [18] define a dental implant as short when it is 8 mm and extra-short implant when an intrabony length is less than 5 mm. However, most of the recent studies refer to implants as short when an intrabony length is 6 mm [11,12,19]

In situations where there is not enough space for a regular implant, a short implant can be considered instead of a regular implant with a maxillary sinus floor augmentation surgery (MSFA). Sinus lift is a procedure that extends treatment (the need for longer healing) and carries the risk of surgical complications. Del Fabbro et al., in the systematic review of long-term implant survival of 6500 implants in 2149 patients in the grafted maxillary sinus, reported the implant survival rate was 93.7% and 97.2% (for lateral approach sinus lift and trans alveolar approach, respectively) [20]. In our group of patients, neither early nor late complications related to the sinus lift procedure were reported. 

Our long-term data have demonstrated a significant difference in survival of our implants between both groups G1 and G2 (87% in short implant compared to 100% in regular implant group). However, this is the longest reported RCT for short implants (6 mm) in lateral maxilla where single non splinted restoration was used. Our data do not comply with the most recently available mid-term five years studies, where the implant survival rate is similar for short and regular implants. In a similarly constructed RCT Thoma et al. has reported the survival rate of short (6 mm) implants at 98.5% and 100% for regular (11–15 mm) implants [19]. Pohl et al., in a three-year follow-up study, reported a 100% survival rate of short implants [21]. A similar long-term short implant survival rate ranging from 86.7% to 100% with a follow-up from one to five years was reported by Papaspyridakos et al. [22]. It is difficult to discuss the results of other authors as different methodologies were adopted, and often, it is not clearly defined what type of restoration was used (single, splinted). The type of reconstruction used is not insignificant. In the studies concerning the short dental implants (≤6 mm length) in three-year observations, the MBL value was found from 1.28 ± 0.37 mm [23] to 0.89 ± 0.25 mm [24]. According to Akram, short implants (<6 mm) in three-year observation period were characterized by MBL of 0.42 mm in the first year of follow-up after loading and significantly reduced to 0.14 mm in the third year as long as PD was slightly reduced from 2.6 mm at the baseline to 2.4 mm during this follow-up [25]. Koldsland, who was investigating the prevalence of peri-implantitis with different degrees of bone loss, in the long follow-up period study (eight years), reported that 23.1% of implants were presented with PD ³4 or ³6 mm [26]. Moreover, 8.2% of the implants in Koldsland study showed a bone loss of 2–3 and 3–4 mm, respectively [26].

Since two implants were lost in G1 compared to zero implants in G2, a case study was conducted over these two cases (one male 45 y and one female 64 y). Both patients regularly once a year reported for follow-up visits for the first three years, and after 36 months, there was no significant deviation from the other mean values in the group, the three-year implant survival rate was 100% for both groups. Interestingly, in both cases in three-year follow-up, a moderate MBL was observed (around 1 mm), and a small amount of HKT was present. Moreover, in both patients, insufficient hygiene and gingivitis symptoms were recorded in the documentation as BOP+. Patients who lost implants were not maintaining their implants in our facility for the last years. From their medical history, it can be found that both of them before the loss of implant, observed gum bleeding during brushing and periodic inflammation in the area of the implant. However, this did not prompt them to visit a specialist. In one case, noticeable implant mobility has prompted patient to visit the dental office where the implant was removed. In the second case implant was removed by the dentist due to severe periimplantitis and later replaced with a regular length implant with sinus lift. In both cases, the presumed cause of implant loss was untreated severe periimplantitis. In our study, the number of noncomplying patients was significantly higher in the group of short implants.

Despite the loss of two implants in short implants, it is worth considering the fact that, in these cases, we were dealing with two noncomplying patients who came for a seven-year follow-up only because of pressure from their surgeon, who was the principal investigator in this study, otherwise they would not report. The remaining parameters during the follow-up study among the implants remaining are similar for both groups. The unequal access to post-operative maintenance can also be considered as a study limitation, this might have resulted in the loss of short implants in non-cooperating patients. This could represent the other limitation and bias in this study to the disadvantage of the group of short implants.

The Consensus report of workgroup 4 of the 2017 World Workshop presents the new definition of periimplantitis as the implant site characterized by inflammation in the periimplant mucosa and subsequent progressive loss of supporting bone [27]. According to metanalysis by Rakic et al., the prevalence of periimplantitis is 18.5% at the patient level and 12.8% at the implant level, so the condition is common [28]. It is worth noting that during the seven-year follow-up, no severe periimplantitis was observed in short implants, whereas in the regular implants group, there were two cases with progressive bone loss >2 mm and BOP+. This finding allows us to conclude that, according to Albrektsson, the success rate was 87% in short implants and 100% in regular implants, and in that group no mobility of the implant, radiolucency, or paresthesia were reported. Wada et al. indicate a clear relationship between a thin zone of keratinized gingiva (<2 mm) and the risk of periimplantis development [29]. There is too little data and the group is too small in our study to assess the critical value for keratinized gingiva.

There are many conditions that can increase the failure rates of the implants, however, according to Papi et al., the relationship between bruxism and dental implant failure is statistically significant [30]. There are also numerous factors that are proven to influence the bone loss around implants. Among them, there are: Platform switching, implant type (bone level or tissue level and the position of the polished implant neck), stability of connection between implant and abutment, and sufficient vertical soft tissue thickness around implant neck. In recent years, a lot of attention in the aspect of MBL marginal bone loss has been paid to the quality and quantity of soft tissues around the dental implant [30,31]. When planning the original research protocol, the STT examination was not taken into account, regardless of the thickness of soft tissues, the implants were placed. During this follow-up, it was decided to extend the original research protocol to examine the thickness of the gingiva around the implants. For this purpose, an ultrasonography examination with a Pirop dental ultrasound device was conducted. The ultrasound device for STT measurement gives accurate and detailed results. Linkevicius et al. [32] found that the initial gingival tissue thickness at the crest may significantly affect MBL around implants. They also found that when soft tissue was less than 2.0 mm thick, more crestal bone loss appeared. Similarly to the other studies [33,34,35], Puzio et al. showed that the thicker the soft tissue, the less MBL is observed, determining a critical value of STT as 2.88 mm and indicating that, in the case of thin soft tissues biotype, gingival augmentation should be done three months before implant placement [16]. However, in our study, the mean STT was 1.78 and 1.79 for G1 and G2, respectively, and only in few cases (30% in G1 and 40% in G2) exceeded the critical value of 2 mm, as defined in the literature, but still, a noticeable negative correlation between MBL and STT was observed, which proves that the thicker soft tissue is, the smaller bone loss can occur. There is too little data and the group is too small in our study to assess the critical value for STT thickness. However, it seems that even a relatively small amount of good quality gingiva around the implant allows for proper daily hygiene and good health of the implant. 

## 5. Conclusions

Our study has demonstrated a lower implant survival rate for short 6 mm implants compared to regular implants (87% compared to 100%). In the case of this study, some issues with the compliance of patients were reported, especially for the group where short implants were lost. This could represent the bias in this study. Despite losing two short implants, good clinical results were achieved in the remaining implants in both groups after the seven-year follow-up period. When considering an overall cost/benefit approach, short implants are a clinically acceptable alternative to regular implants with a sinus lift surgery.

## Figures and Tables

**Table 1:** Patient demographics.

Demographics of the Study Population				
	Short Implants (G1)	Regular Implants (G2)	Total	*p*-Value
Number of implants (Baseline)	15	15	30	
Number of implants (seven years)	13	15	27	
Implants survived (seven years)	87%	100%	93%	0.4828 Fisher’s exact test
Age in years at base line mean ± SD (Range)	48.8 ± 11.4 (26–64)	42.3 ± 13.5 (26–63)	45.5 ± 12.7 (26–64)	0.1623 unpaired *t* test
Age in years after seven years mean ± SD (Range)	54.9 ± 11.4 (33–70)	49.3 ± 13.5 (33–70)	51.9 ± 12.7 (33–70)	0.2451 unpaired *t* test
Gender	10 Females, 5 Males	10 Females, 5 Males		
Implant length	6 mm	11 and 13 mm		

**Table 2:** Clinical outcome measures.

		Short Implants G1	Regular Implants G2	*p*-Value
HKT (mm)	Mean:	2.95	2.55	0.4126 unpaired *t* test
	SD:	1.42	1.18	
	Range:	1.0–6.0	1.0–5.0	
PD (mm)	Mean:	2.62	2.63	0.9456 unpaired *t* test
	SD:	0.51	0.81	
	Range:	2.0–3.0	1.0–5.0	
STT (mm)	Mean:	1.78	1.79	0.9497 unpaired *t* test
	SD:	0.58	0.61	
	Range:	1.0–2.6	0.9–2.5	
BOP	No %	53.85%	40.00%	0.7051 Fisher’s exact test
	Yes %	46.15%	60.00%	

**Table 3:** Crown-to-implant ratio (*C*/*I* ratio), * significant different.

		Short Implants G1	Regular Implants G2	*p*-Value
C/I ratio	Min	1.36	0.68	<0.0001 * unpaired *t* test significant different
	Max	1.97	1.65	
	Mean:	1.64	1.06	
	SD:	0.20	0.29	
	Median:	1.68	1.05	

**Table 4:** Marginal bone loss.

		Short Implants G1	Regular Implants G2	*p*-Value
MBL seven years	Min	0.10	0.00	0.3263 Mann–Whitney U
	Max	1.30	2.20	
	Mean:	0.50	0.53	
	SD:	0.35	0.75	

**Table 5:** Correlations.

Correlation	C/I Ratio: MBL	MBL:HKT	MBL:STT	HKT:STT
r	−0.02666	−0.1548	−0.3899	0.5201
Test	Spearman	Spearman	Spearman	Pearson
Significance	No	No	Yes (negative correlation)	Yes (positive correlation)

## Data Availability

The datasets analyzed during the current study are not publicly available but are available from the corresponding author on reasonable request.
